# Acute effect of vigorous aerobic exercise on the inhibitory control in adolescents

**DOI:** 10.1016/j.rppede.2016.01.005

**Published:** 2016

**Authors:** Rodrigo Alberto Vieira Browne, Eduardo Caldas Costa, Marcelo Magalhães Sales, André Igor Fonteles, José Fernando Vila Nova de Moraes, Jônatas de França Barros

**Affiliations:** a Universidade Federal do Rio Grande do Norte (UFRN), Natal, RN, Brazil; b Universidade Católica de Brasília (UCB), Brasília, DF, Brazil; c Universidade Federal do Vale do São Francisco (Univasf), Petrolina, PE, Brazil

**Keywords:** Sports, Physical education and training, Cognition, Executive function, Puberty

## Abstract

**Objective:**

To assess the acute effect of vigorous aerobic exercise on the inhibitory control in adolescents.

**Methods:**

Controlled, randomized study with crossover design. Twenty pubertal individuals underwent two 30-minute sessions: (1) aerobic exercise session performed between 65% and 75% of heart rate reserve, divided into 5 min of warm-up, 20 min at the target intensity and 5 min of cool down; and (2) control session watching a cartoon. Before and after the sessions, the computerized Stroop test-Testinpacs™ was applied to evaluate the inhibitory control. Reaction time (ms) and errors (*n*) were recorded.

**Results:**

The control session reaction time showed no significant difference. On the other hand, the reaction time of the exercise session decreased after the intervention (*p*<0.001). The number of errors made at the exercise session were lower than in the control session (*p*=0.011). Additionally, there was a positive association between reaction time (*Δ*) of the exercise session and age (*r* ^2^=0.404, *p*=0.003).

**Conclusions:**

Vigorous aerobic exercise seems to promote acute improvement in the inhibitory control in adolescents. The effect of exercise on the inhibitory control performance was associated with age, showing that it was reduced at older age ranges.

## Resumo

**Objetivo:**

Verificar o efeito agudo do exercício aeróbio vigoroso sobre o controle inibitório em adolescentes.

**Métodos:**

Estudo controlado e randomizado com delineamento cruzado. Vinte púberes foram submetidos a duas sessões de 30 minutos: 1) sessão exercício aeróbio feito entre 65-75% da frequência cardíaca de reserva, com cinco minutos para aquecimento, 20 minutos na intensidade alvo e cinco minutos de volta à calma; e 2) sessão controle assistindo a desenho animado. Previamente e após as sessões, o teste de Stroop computadorizado (Testinpacs^®^) foi aplicado para avaliar o controle inibitório. O tempo de reação (ms) e os erros cometidos (n) foram registrados.

**Resultados:**

O tempo de reação da sessão controle não apresentou diferença significativa. Por outro lado, o tempo de reação da sessão exercício diminuiu após a intervenção (*p*<0,001). Os erros cometidos na sessão exercício foram menores do que na sessão controle (*p*=0,011). Adicionalmente, houve associação positiva do tempo de reação (Δ) da sessão exercício com a idade (*r* ^2^=0,404; *p*=0,003).

**Conclusões:**

O exercício aeróbio vigoroso parece promover melhoria aguda no controle inibitório em adolescentes. O efeito do exercício sobre o desempenho do controle inibitório foi associado à idade e demonstrou ser reduzido em faixas etárias mais altas.

## Introduction

Executive control (or executive functions) refers to higher cognitive processes that manage the control of other, more basic cognitive functions and direct the ideal behavior to achieve goal-oriented behaviors.^1^ In general, executive control is subdivided into inhibitory control (IC), working memory and cognitive flexibility.^1^ The IC is considered the main domain of executive control and a determinant of academic success, as it controls attention, behavior, thought and/or emotion to override a strong internal predisposition or external attraction and adapt itself to conflicting situations.^1^

School activities constitute a model of environmental request regarding the autonomy and control of attentional, organization and planning functions, which requires an efficient performance of the IC.^2^ Evidence suggests that the development of IC skills during childhood promotes an increased capacity for success in the development of the theory of mind - facilitates thinking and learning^3^ - as well as a better performance in counterfactual^4^ reasoning and strategic^5^ tasks. The IC also has been strongly associated with intelligence level^6^ and school performance.^7^

Both frontal regions, cortical and subcortical, subserve the executive control.^8^ The prefrontal cortex (PFC) is the one that plays a key role.^8^ Increased brain activity of the PFC was observed during the task making of the IC (Stroop Test).^9^ The PFC comprises from a quarter to a third of the cerebral cortex and contains rich reciprocal connections with itself, with other cortical areas and subcortical and limbic regions.^10^ The performance of executive control develops from early childhood, throughout adolescence, to adulthood,^11^ concurrently with the neuroanatomical, functional^12^ and brain blood perfusion^13^ changes, including the PFC regions.

Physical exercise has been considered an important environmental factor for neurodevelopment,^14^ to promote cognitive and brain health,^15^ as well as for a better performance of the executive and school control.^7^ A single session of aerobic exercises has been shown to improve the efficiency of the IC in children^16^ and young adults,^17^^-^ 19 in contrast to what was observed in adolescents after 20 min of aerobic exercise performed on a cycle ergometer at 60% of maximum heart rate (HR_max_).^20^ Cognitive performance after acute exercise seems to be dependent on the intensity.^21^ In the meta-analysis of Chang et al.,^21^ the studies that used low intensity, <50% of HR_max_, had a negative effect on cognitive performance. On the other hand, in studies with intensities >64% of HR_max_, the effects were positive.

A possible physiological hypothesis that may explain the acute effect of exercise intensity on the IC is increased cerebral blood flow generated by exercise effort, which can have an impact on post-exercise cognitive performance.^17^^-^ 19 In the study by Yanagisawa et al.,^19^ there was an increase in cerebral blood flow (↑ oxygenated hemoglobin) in the PFC and improved performance in the Stroop test in young adults after 10 min of aerobic exercise at 50% of the peak oxygen consumption (peak VO_2_). The same effect was observed in similar experiments carried out with young adults after 20 min of exercise between 60% and 70% of HR_max_ 18 and after 15 min of exercise at 40% of maximum load, respectively,^17^ and in children after 20 min of aerobic exercise between 65% and 75% of HR_max_.^16^

However, there is still a gap of knowledge whether a vigorous aerobic exercise session can improve the IC in adolescents, which may be important, as their PFC is still undergoing the maturation phase. It is a period of structural, functional^12^ and blood perfusion^13^ changes. The study hypothesis is that the use of a prescription of aerobic exercise with control of the intensity, volume and other confounding factors associated with cognitive improvement by acute exercise, may favor the physiological brain mechanisms induced by exercise and influence the post-exercise IC performance, as shown in children^16^ and young adults.^17^^-^ 19 Therefore, the aim of this study was to investigate the acute effect of vigorous aerobic exercise on the IC in adolescents. The intensity and volume of the exercise were prescribed according to vigorous exercise recommendations for adolescents from the World Health Organization^22^ and according to the best effect obtained by meta-analysis of Chang et al.^21^ Other control factors, such as time of the exercise and the time interval to apply the post-exercise cognitive test, were also supported by the meta-analysis of Chang et al.^21^

## Method

This was a controlled, randomized study with crossover design, carried out on the seaside, in the municipality of Icapuí (CE). The acute effect of the exercise prescription protocol was tested in two sessions, with a minimum of 48 h of interval, namely: (1) vigorous aerobic exercise session; and (2) control session watching an age-appropriate cartoon. Half of the participants, randomly, first received the experimental treatment and then the control, while the other half first received the control and after the experimental treatment. Finally, the assessment of IC performance was performed before and after the sessions.

Sample size was calculated using the statistical power (1 − *β*), with analysis of variance being used in the main study outcome (Split Plot ANOVA), with an effect size of *f*=0.333 (considered middle-sized) and an alpha of 0.05. The statistical power given to this sample, regardless of gender, was 80% (G*Power^®^, version 3.1.9.2; Institute for Experimental Psychology in Dusseldorf, Germany).

Twenty adolescents (Table 1) of both genders and physically active, between 10 and 16 years of age, were randomly enrolled from public elementary schools in the municipality of Icapuí. Posters about the study were fixed in the bulletin boards of schools to recruit volunteers and a lecture was given at a predetermined time and location for those interested. Inclusion criteria were: (i) availability to attend the initial assessment and the control and exercise sessions in the morning; (ii) to be physically active, that is, to be enrolled and regularly practice physical exercises (≥1 year and ≥2×/week, respectively) in extracurricular sports programs taking place in the schools in the shift opposite to the school shift; (iii) meet the criteria of the physical activity readiness questionnaire (PAR-Q); (iv) to be classified as “pubertal” (Tanner stages 2-4)^23^; and (v) have no physical or intellectual disabilities and/or clinical, neuromotor, psychological and/or cognitive contraindications.

**Table 1:** Characterization of the sample of adolescents (*n* =20). Data expressed as mean and standard deviation for parametric variables, median and 95% confidence intervals for nonparametric variables and range (minimum-maximum).

	Proportion, measures of position and dispersion	Minimum-maximum
Gender (male/female)	11/9	
Maturation (stages: 2/3/4)	7/6/7	
Age (years)	13.0±1.8	(10.0-16.0)
Body mass (kg)	51.4±9.3	(34.0-76.0)
Height (cm)	157.4±9.5	(146.0-180.0)
BMI (kg·m^-2^)	20.6±2.3	(15.4-24.5)
VO_2max_ (mL·kg^-1^·min^-1^)^a^	48.4 (47.4-51.8)	(43.9-59.9)
*V*_max_ (km·h^-1^)	11.6±1.2	(9.5-14.0)
HR_max_ (bpm)	203.6±6.0	(192.0-213.0)
HRrest (bpm)	65.0±7.3	(50.0-76.0)
HRcontrol (bpm)	80.8±7.9	(67.3-99.8)
HRexercise (bpm)	165.4±8.3	(149.8-176.8)
HRR (%)^a^	73.5 (70.0-73.3)	(65.0-75.0)

HRcontrol, mean heart rate of the control session; HRexercise, mean heart rate of the exercise session; HR_max_, maximum heart rate; HRR, percentage of heart rate reserve at the exercise session; HRrest, resting heart rate; BMI, body mass index; *V*_max_, maximum velocity; VO_2max_, maximal oxygen uptake.

a Nonparametric variable.

The research project was approved by the Institutional Review Board of Universidade Federal do Rio Grande do Norte (Protocol No. 876286/2014 CEP/UFRN), consistent with the Declaration of Helsinki and Resolution N. 466/2012 of the National Health Council. All selected adolescents had the informed consent and assent form, PAR-Q and medical-history questionnaire filled out and duly signed.

Body weight and height were measured by a mechanical scale (G-Tech^®^) and a stadiometer fixed to the wall (Sanny^®^), respectively. The body mass index (BMI) was calculated through the formula: [BMI=body mass (kg) × height (m)^2^].^24^

Sexual maturation was evaluated by Tanner stage^23^ to differentiate pre-pubertal individuals from pubertal ones, as inclusion criteria in the study.^25^ The Tables consist of images with captions that characterize the genitals and body hair for boys and breasts and body hair for girls. The method and its purpose were individually explained and, through self-assessment, the adolescents pointed out in a private form which images their condition most resembled. The pubertal stage was rated on a scale from 1 to 5, namely: 1=prepubertal; 2-4=pubertal; and 5=post-pubertal.

The maximal multistage 20-m shuttle run test^26^ was applied to obtain the HR_max_. This test was performed in an indoor sports court (between 7 and 10 h) and consists of racing back and forth at a distance of 20 meters. The velocity was controlled by a metronome audio. The initial velocity was 8.5 km/h, followed by increments of 0.5 km/h at every one-minute stage. Heart rate (HR) was monitored throughout the test by a heart rate monitor (Beurer^®^, Germany). HR_max_ was considered as the highest HR attained during the test, valid only when signs of intense effort were observed.^27^ Subjects were verbally motivated to endure as long as possible. The test was continued until voluntary exhaustion. The maximum velocity (*V*_max_) was used to estimate the maximum oxygen consumption (VO_2max_) using Léger et al. equation.^28^

The resting heart rate (HRrest) was measured with the subject at rest in the supine position for 5 min. It was considered the lowest HR obtained. The HR reserve (HRR) was the difference between HR_max_ and HRrest (HRR=HR_max_ − HRrest). To determine the HR-target for exercise prescription, we used the percentage of HRR. The percentage of HRR was added to HRrest to determine the HR-target in exercise: HR-target = (%intensity (in decimal) × HRR) + HRrest.^24^

On a different day of the exercise session, subjects were familiarized with exercise intensity and how to stay on the HR-target. At the exercise session, a warm-up of 5min was performed through walking, followed by 20min of running at vigorous intensity (65-75% of HRR) and 5 min of cooling down, totaling 30 min. The HR was monitored throughout the session by a heart rate monitor (Beurer^®^, Germany), so that the participant remained in the HR-target. In addition to the individual control of the participants themselves, according to the guidelines given during the familiarization session, the HR was monitored and recorded every 3min. If necessary, verbal directions were provided to adjust the intensity. The exercise was performed on the beach sand (wet sand, low tide and flat terrain), with the subjects barefoot. The sessions were carried out between 7 and 10 AM with a temperature and air humidity between 26-30 °C and 52%-74%, respectively.

During the control session, the subjects remained seated for 30 min in the school computer lab watching an age-appropriate cartoon (Kung Fu Panda). HR was monitored and recorded every 3 min by a heart rate monitor (Beurer^®^, Germany).

The IC performance was evaluated by the computerized Stroop test (Testinpacs^®^), with the aid of a desktop and a 14-inch monitor.^29^ Recalling was done before every session. The test was applied before and after 10 min of each session. The instrument has three Phases, the first two congruent and the last one incongruent. The index and middle fingers of the right hand remained on the left (←) and right (→) arrow keys, respectively, which were activated according to each stimulus. In stage 1, rectangles in green, blue, black and red were individually shown in the center of the monitor. In the lower corners of the monitor, answers corresponding or not to the rectangle color were exhibited until the participant responded to the attempt by pressing the keys ← or →. In stage 2, both stimuli and responses were shown as words, always in white color. The response was considered correct when the stimulus and response coincided. Finally, in stage 3, the word of one of the four colors was exhibited in incompatible color. The subject was instructed to press the key corresponding to the color of the word and inhibit the response to the word identity. At all stages the stimuli were presented automatically and randomly, 12 attempts per stage. The reaction time (RT) in milliseconds (ms) and the number of errors (*n*) made at each stage were recorded.

The Statistical procedures were performed with SPSS for Win/v.19.0 (Statistical Package for Social Sciences, Chicago, IL, USA) and G* Power version 3.1.9.2 (Institute for Experimental Psychology in Dusseldorf, Germany). The normality and homogeneity of data variance were tested by the Shapiro-Wilks test and Levene test, respectively. Parametric variables were expressed as mean and standard deviation or standard error, and the nonparametric as mean or median and their respective 95% confidence intervals. The level of significance was set at *p*<0.05.

The reliability of the Stroop test (RT) between baseline values (pre × pre) was assessed by Cronbach's alpha (*α*). The *t* test for independent samples was used to compare the baseline RT between the conditions (pre × pre). Split-Plot Anova, adjusted for chronological age, was applied to the comparison of intra- and inter-conditions of RT. The hypothesis of sphericity was verified by Mauchly test and, when violated, the degrees of freedom are corrected by the Greenhouse-Geisser estimates. The effect size of the variance was calculated by the eta squared (*η* ^2^). The paired *t*-test was applied to each group separately, in order to locate the differences observed in the Split-Plot. The size effect was calculated by the equation: 


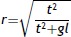


Quade's Non-parametric Ancova, adjusted by chronological age, was applied to compare the deltas (*Δ*=pre-post) of the amount of errors made between the conditions (control × exercise). After the assessment of normality and homogeneity of the variance residues, the simple linear regression (*r* ^2^) and Pearson's correlation coefficient (*r*) were applied to associate the RT delta (*Δ*=post-pre) of the incongruent Phase 3 with chronological age.

## Results

Among the adolescents selected for the study, it was observed that 65% had failed a school year. Table 1 shows the variables of the sample characteristics, sexual maturation, anthropometric measurements, estimated variables and those obtained by the maximal multistage 20-m shuttle run test (*V*O_2max_, *V*_max_ and HR_max_) and mean HR of the control and exercise sessions. Furthermore, the HRR% mean at which the adolescents remained during the exercise session was depicted.

The RT of the stages 1, 2 and 3 of the Stroop test between baseline values (pre × pre) had a reliability of *α*=0.502; *α*=0.493; *α*=0.752; respectively. There was no difference between the baseline values of RT (pre × pre) in stage 1 (*t*[38]=−0.567; *p*=0.574), in stage 2 (*t*[38]=−0.740; *p*=0.464) and stage 3 (*t*[38]=−0.665; *p*=0.510).

**Figure 1:**
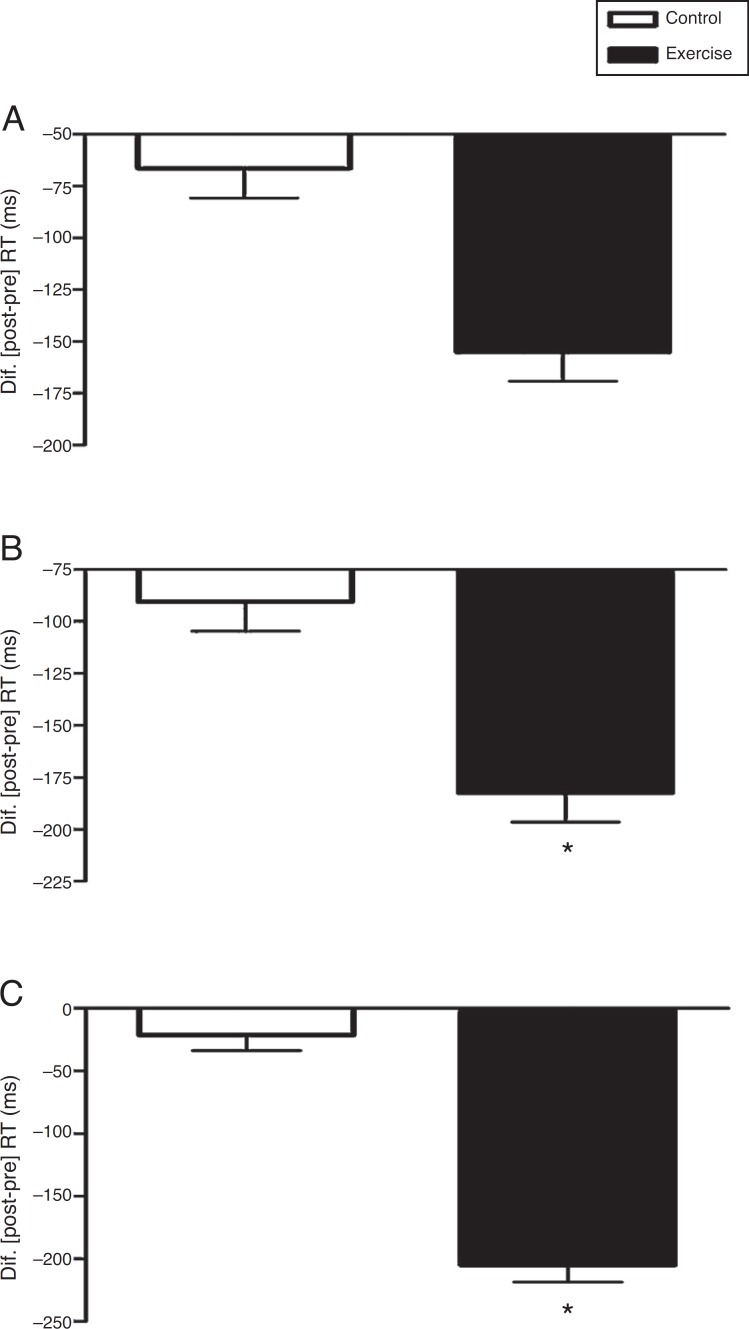
Acute effect of vigorous aerobic exercise on the reaction time (RT) in Phases 1 (A), 2 (B) and 3 (C) of the computerized Stroop Test (Testinpacs^®^). Split-Plot Anova adjusted for chronological age was applied in the intra- and inter-comparisons between conditions (2×2). The delta data (*Δ* = post-pre) are shown as mean and standard error. *p<0.001; pre × post.

In the congruent phase 1 of the Stroop test, the interaction time×conditions, adjusted for age did not differ statistically, *F*(1,37)=1.98; *p*=0.168; *η* ^2^=0.051. There was no difference between the conditions, *F*(1,37)=0.00; *p*=0.982; *η* ^2^=0.000. There was also no significant effect on time, *F*(1,37)=2.79; *p*=0.103; *η* ^2^=0.070 (Fig. 1A).

The second congruent phase, stage 2, did not show statistical difference in the time×conditions interaction, adjusted for age, *F*(1,37)=2.28; *p*=0.139; *η* ^2^=0.058. There was no difference between the conditions, *F*(1,37)=0.00; *p*=0.955; *η* ^2^=0.000. There was a significant effect on time, *F*(1,37)=4.66; *p*=0.037; *η* ^2^=0.112. The paired *t* test (pre×post) showed that the control condition did not differ, *t*(19)=2.05; *p*=0.055; *r*=0.43; but the exercise condition significantly differed, *t*(19)=4.20; *p*<0.001; *r*=0.69 (Fig. 1B).

**Figure 2:**
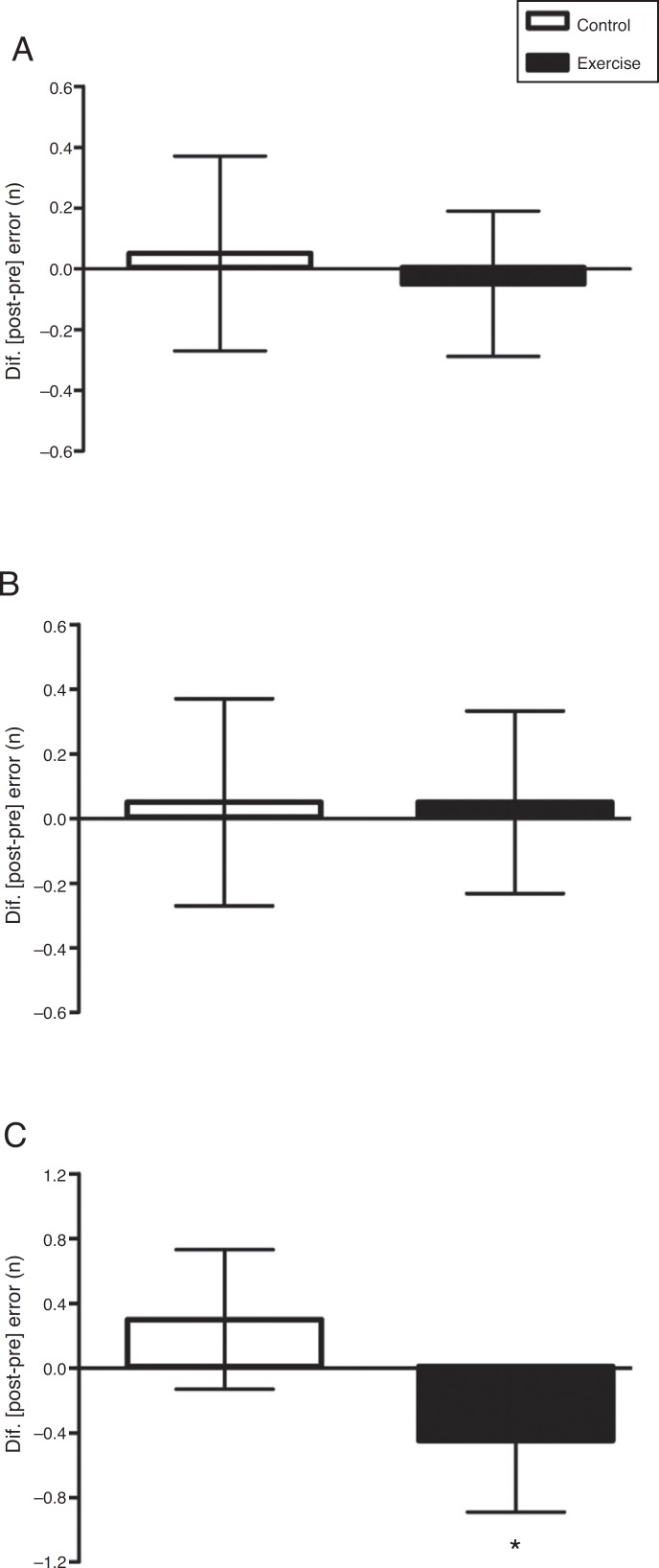
Acute effect of vigorous aerobic exercise on the errors made in Phases 1 (A), 2 (B) and 3 (C) of the computerized Stroop Test (Testinpacs^®^). Non-parametric Ancova adjusted for chronological age was applied in the comparison between conditions. The delta data (*Δ* = post-pre) are shown as mean and 95% confidence interval (95%CI). *p<0.011; control × exercise.

In the incongruent phase, stage 3, there was an interaction time×conditions that was statistically significant, adjusted by age, *F*(1, 37)=12.49; *p*=0.001; *η* ^2^=0.252. There was no difference between the conditions, *F*(1, 37)=0.134; *p*=0.716; *η* ^2^=0.004. There was a significant effect on time, *F*(1, 37)=5.64; *p*=0.023; *η* ^2^=0.132. The paired *t* test (pre×post) showed that the control condition did not differ, *t*(19)=0.64; *p*=0.532; *r*=0.15; but the exercise condition significantly differed, *t*(19)=4.94; *p*<0.001; *r*=0.75 (Fig. 1C). The errors that were made, adjusted by age, did not differ between the control × exercise conditions in phase 1 [*F*(1, 38)=0.105; *p*=0.748 (Fig. 2A)] and phase 2 [*F*(1, 38)=0.045; *p*=0.834 (Fig. 2B)], but phase 3 showed a significant difference [*F*(1, 38)=7.162; *p*=0.011 (Fig. 2C)].

**Figure 3:**
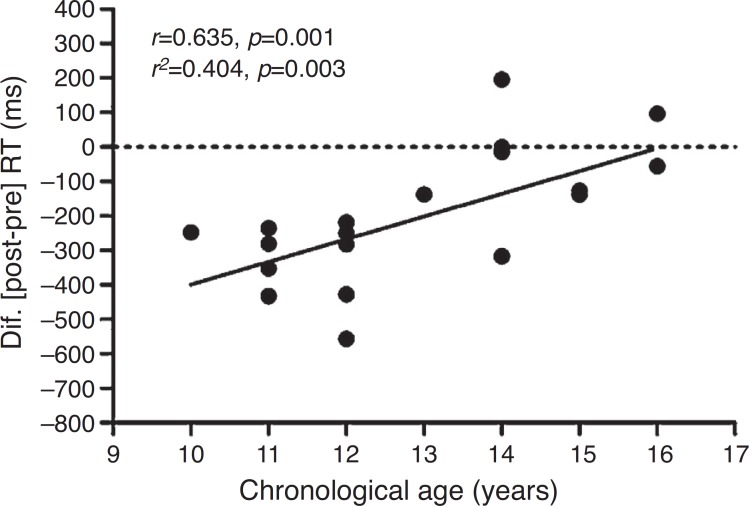
Linear regression (*r* ^2^) and Pearson's coefficient correlation (*r*) between the delta (*Δ*=post-pre) of the incongruent reaction time (RT) of Stroop test Phase 3 of the exercise session with chronological age.

As secondary analysis, we verified a positive association (*p*=0.003) of ΔRT of incongruent phase 3 of the exercise condition with chronological age (Fig. 3). On the other hand, there was no association of ΔRT of phase 3 of the control condition with chronological age (*r* ^2^=0.021; *p*=0.545). Sample size calculation, afterwards, of the analysis of association was carried out with an alpha of 0.05, sample size of 20 subjects and a coefficient of correlation of 0.635. The statistical power given in the analysis was 88%.

## Discussion

The present study investigated the acute effect of vigorous aerobic exercise on the IC in adolescents. The main findings suggest that vigorous aerobic exercise acutely enhances the performance of the IC, as there was an improvement on the Stroop interference (inhibition of response) observed by the decrease in the RT of the incongruent phase (Fig. 1C), with a focal effect considered large (*r*=0.75), accompanied by a smaller number of errors made (Fig. 2C). These results can be very important to elucidate the influence of exercise on the IC efficiency and, therefore, contribute to the process of learning in the school environment.^2^

The influence that exercise promoted on IC performance is in accordance with the evidence from studies carried out with children^16^ and young healthy adults.^17^^-^ 19 As exercise intensity is an important factor to enhance post-exercise cognitive performance,^21^ this study adopted a vigorous exercise protocol, which in addition to being recommended by the World Health Organization for adolescents,^22^ would be adequate to induce the physiological mechanisms responsible for favoring the cognitive performance.^21^ For instance, in a meta-analysis, the intensity of the exercise had a significant influence so that the prescribed exercise <50% of HR_max_ resulted in a significant negative effect (*d*=−0.113) on cognitive performance, but when prescribed at 64-76% or 77-93% of the HR_max_, the results were positive, with an effect of 0.202 and 0.268, respectively.^21^

In addition to exercise intensity, other controlled factors may have been equally important for the exercise to favor the cognitive benefits, including the volume and time of the exercise, as well as the time interval for the implementation of the post-exercise cognitive test. In this sense, the exercise volume was also adjusted according to the recommendations of the World Health Organization^22^ on the volume of vigorous exercise for adolescents and, as other studies have shown, resulting in cognitive benefits after 20 min of aerobic exercise in children^16^ and young adults.^18^

The time interval to apply the cognitive test was defined between 10 and 20 min post-exercise, according to the best effect size obtained by the meta-analysis of Chang et al.,^21^ in which this control factor was considered a primary moderator of cognitive benefits. For instance, Chang et al.^21^ categorized the time to apply the cognitive tests in 0-10 min; 11-20 min or >20 min after the exercise and obtained an effect size of −0.060, 0.262 and 0.171 respectively. That is, the post-exercise application time significantly influenced the effect size. The positive effects were observed only after 11 min post-exercise.

The statistical findings of the meta-analysis by Chang et al.^21^ on the time interval for the application of the post-exercise cognitive tests is consistent with reticular-activating hypofrontality model proposed by Dietrich and Audiffren,^30^ which suggests a decrease in brain activation in regions not directly associated with physical exercise (i.e., PFC) to supply directly associated regions. Corroborating this understanding, the study by Wang et al.^31^ showed a decline in IC performance during vigorous aerobic exercise, which could influence IC efficiency immediately after the exercise. Thus, these studies and this theoretical model agree with the need for a time interval between the end of the exercise and the beginning of a cognitive task for homeostasis to occur at the brain level, more specifically in the PFC.

Our data show that aerobic exercise seems to have been a tool capable of acutely influencing the physiological mechanisms responsible for favoring IC performance. The mechanisms are still somewhat controversial and little explored, but the main physiological hypothesis that could explain these effects refer to the increase in cerebral blood flow, which can influence cognitive performance after exercise.^17^^-^ 19 For instance, Yanagisawa et al.,^19^ using near-infrared spectroscopy (NIRS), evaluated the cortical activation during Stroop test performed before and after 10 min of aerobic exercise at 50% of peak VO_2_ in 20 young adults. In fact, there was increased blood flow from the lateral PFC in both hemispheres (↑oxygenated hemoglobin) due to Stroop interference (incongruence). In contrast, this activation was significantly increased in the left dorsolateral PFC and coincided with improved performance on the cognitive task (↓RT).

As secondary analysis, we demonstrated an association of ΔRT of the incongruent phase (stage 3) of the exercise condition with chronological age (*r* ^2^=0.404) (Fig. 3). That is, post-exercise IC improvement was reduced in older age groups. The regression analysis shows that 40.4% of the variability found in post-exercise IC performance can be explained by chronological age. In this sense, due to evidence that one of the physiological mechanisms responsible for the improvement of post-exercise IC can be increased cerebral blood flow,^17^^-^ 19 the variability in Δ can be related to the maturation of the cerebral perfusion mechanism during adolescence.^13^ Notably, Satterthwaite et al.^13^ observed a significant impact of puberty on cerebral perfusion development. The authors evaluated the cerebral blood flow of 922 young individuals aged eight to 22 years and found that, during adolescence, the brain flow throughout the cortex decreases considerably, including in the PFC. The cerebral blood flow of the middle gray matter undergoes a sharp decline in late childhood and early adolescence until about 16-18 years, followed by a slight increase during early adulthood.

Although original this study has limitations, one of which is the fact that it did not assess the IC performance combined with a neuroimaging technique, which could promote greater support in confirming the evidence. Nevertheless, several studies have confirmed that acute aerobic exercise can improve IC at the macroneural level, as it promotes significant impact on PFC brain activity during IC task making after 10-20 min of aerobic exercise in children^16^ and young adults.^17^^,^ 18

The use of sexual maturation self-assessment procedure to distinguish the pre-puberty and puberty as an inclusion criterion is also considered a limiting factor. The evaluation by visual inspection by a trained evaluator is the most reliable indirect method.^25^ However, it can be justified that the sample consisted mostly of adolescents at the pubertal stage.^25^ In the study by Rasmussen et al.,^25^ carried out with 898 children and adolescents, the self-assessment of sexual maturation showed to be a sufficiently accurate technique to simply differentiate simples between prepubertal and pubertal stages. Breast stage was correctly assessed by 44.9% of the girls (*κ*=0.28; *r*=0.74; *p*<0.001) and the genital stage by 54.7% of boys (*κ*=0.33; *r*=0.61; *p*<0.001). Pubic hair was correctly assessed by 66.8% of girls (*κ*=0.55; *r*=0.80; *p*<0.001) and 66.1% of boys (*κ*=0.46; *r*=0.70; *p*<0.001).

It is noteworthy that this study is a pioneer in investigating the acute effects of vigorous aerobic exercise on IC in adolescents and contributes to elucidate the effects of aerobic exercise in this population, which have an executive control performance^11^ and brain functions, structures^12^ and perfusion^13^ still undergoing maturation. Additionally, the study had the premise of controlling the possible variables, such as the equal number of adolescents of both genders, obtaining the HR_max_ through a maximal effort progressive running test, the intensity and volume of the experimental condition exercise, the time of physical exercise, the use of a computerized cognitive test that records the performance in milliseconds, and the time for application of the post-exercise cognitive test. Therefore, the study may provide subsidies for the applicability of this aerobic exercise protocol in the school context.^32^

In conclusion, a vigorous aerobic session performed for 20 min seems to promote improvement in IC capacity in adolescents. Additionally, the effect of exercise on the IC performance was associated with chronological age. This demonstrates that the benefits of exercise were reduced in older age groups. As practical implications, the findings of this study contribute to justify the inclusion of physical education classes during school hours, that is, among the other school subjects (i.e., Portuguese and mathematics), as well as the inclusion of a physically active interval/recess, as 20 min of vigorous aerobic exercise can favor the IC efficiency and therefore contribute to learning improvement after 10 min of post-exercise recovery.
